# Metabolomic profiling reveals candidate biomarkers and key metabolic pathways associated with milk production performance in Sapera dairy goats

**DOI:** 10.14202/vetworld.2026.3109-3124

**Published:** 2026-07-20

**Authors:** Rohmiyatul Islamiyati, Athhar Manabi Diansyah, Rahmat Rahmat, Aeni Nurlatifah, Ismah Ulfiyah Azis, Fahrul Irawan, Andi Muhammad Alfian

**Affiliations:** 1 Faculty of Animal Science, Hasanuddin University, Jl. Perintis Kemerdekaan Km. 10 Tamalanrea Makassar, South Sulawesi, 90245, Indonesia.; 2 Faculty of Agriculture, Lambung Mangkurat University, Jl. A. Yani Km 36, Banjarbaru, South Kalimantan, 70714, Indonesia.; 3 Department of Nutrition and Feed Technology, Faculty of Animal Science, Gadjah Mada University, Depok, Sleman Regency, Special Region of Yogyakarta, 55281, Indonesia.

**Keywords:** amino acid metabolism, biomarkers, dairy goats, lactation efficiency, lipid metabolism, metabolomics, milk production, Sapera goats

## Abstract

**Background and Aim:**

Milk production efficiency in dairy goats is strongly influenced by metabolic adaptation during lactation. However, information regarding metabolomic signatures associated with milk production performance in Sapera goats remains limited. This study aimed to characterize serum metabolomic differences between high-production (HP) and low-production (LP) Sapera goats and to identify candidate biomarkers and metabolic pathways associated with milk production performance using a liquid chromatography-high resolution mass spectrometry (LC-HRMS)-based metabolomics approach.

**Materials and Methods:**

Twenty lactating Sapera goats were classified into HP (n = 10) and LP (n = 10) groups according to previously established milk yield records. Blood samples were collected from the jugular vein, and serum metabolites were profiled using LC-HRMS. Principal component analysis (PCA) and partial least squares-discriminant analysis (PLS-DA) were used to evaluate metabolic differences between groups. Differential metabolites were identified based on statistical significance and variable importance in projection scores. Metabolite set enrichment and Kyoto Encyclopedia of Genes and Genomes pathway analyses were subsequently performed to identify biological pathways associated with differences in production.

**Results:**

Both PCA and PLS-DA demonstrated clear separation between HP and LP goats, indicating distinct metabolic signatures. Fifteen discriminant metabolites were identified as candidate biomarkers associated with milk production performance. Metabolites related to energy and lipid metabolism, including pyruvate, fumarate, glycerol 3-phosphate, β-hydroxybutyric acid, and 3-hydroxy-3-methylglutaric acid, were more abundant in HP goats. In contrast, sphinganine, phytosphingosine, sphingosine, cystathionine, and ceramide-related intermediates were enriched in LP goats. Enrichment and pathway topology analyses revealed that fatty acid degradation, fatty acid elongation, the citrate cycle, carbohydrate metabolism, and amino acid metabolism were the principal pathways associated with differences in milk production.

**Conclusion:**

Distinct systemic metabolic signatures were associated with milk production performance in Sapera goats. The identified metabolites indicate coordinated alterations in lipid, central carbon, and amino acid metabolism and may serve as candidate biomarkers of lactation efficiency. These findings provide valuable insights for precision nutrition and metabolic management strategies in dairy goat production systems. Further validation using larger populations and longitudinal studies is required to confirm their predictive utility.

## INTRODUCTION

Milk production is one of the most economically important traits in dairy goat production systems, particularly in high-yielding breeds such as Sapera goats. Sapera goats, developed through crossbreeding between Saanen and Etawah goats, are widely used as dairy goats in Indonesia because they combine relatively high milk production potential with adaptation to tropical production environments. Tropical environmental conditions may further influence lactation metabolism by affecting feed intake, energy balance, and physiological adaptation. Increasing global demand for dairy products has intensified the need to improve productivity and efficiency in dairy livestock while maintaining animal health and metabolic stability [1]. Milk yield is a complex trait influenced by multiple factors, including genetics, nutrition, physiological status, and metabolic regulation. Among these factors, metabolic adaptation plays a crucial role in determining lactating animals' ability to sustain high milk production. During lactation, animals experience substantial metabolic demands because large amounts of energy and nutrients are required to synthesize milk components such as lactose, milk fat, and milk proteins [2]. Therefore, understanding the metabolic mechanisms underlying variation in milk production is essential for improving dairy goat productivity and developing more effective management strategies.

Recent advances in metabolomics have provided powerful tools for investigating metabolic processes associated with animal productivity and physiological performance. Metabolomics enables comprehensive profiling of small-molecule metabolites that reflect an organism's biochemical state and provide insights into metabolic pathways that regulate physiological functions [3]. Because metabolites represent downstream products of gene expression and enzymatic activity, metabolomic analysis can capture functional changes in metabolic networks associated with physiological traits such as lactation performance [4]. In livestock research, metabolomics has increasingly been applied to identify metabolic biomarkers associated with milk production, metabolic efficiency, disease resistance, and nutritional status [5].

In dairy animals, lactation requires coordinated metabolic regulation of lipid, carbohydrate, and amino acid metabolism to meet the energetic and biosynthetic demands of milk synthesis. Lipid metabolism plays a critical role in providing energy substrates and structural components required for milk fat synthesis [6]. Carbohydrate metabolism, particularly glucose production through gluconeogenesis, is essential for lactose synthesis in the mammary gland, which ultimately determines milk volume [7]. Amino acid metabolism also contributes to milk protein synthesis and to metabolic signaling pathways that regulate nutrient utilization and cellular metabolism [8]. Consequently, alterations in these pathways may reflect physiological adaptations associated with different levels of milk production.

Despite the growing use of metabolomics in dairy animals, breed-specific information on serum metabolic signatures associated with differences in milk production remains limited in Sapera goats. Previous studies have mainly focused on dairy cattle or on general metabolic adaptations during lactation, whereas comprehensive characterization of serum metabolites associated with contrasting milk production phenotypes has received little attention in Sapera goats. Moreover, studies integrating biomarker discovery with metabolite enrichment and pathway topology analyses are scarce in this breed. Consequently, the biochemical mechanisms underlying variation in lactation efficiency and the metabolic pathways supporting superior milk production remain incompletely understood. Identification of such metabolic signatures could provide valuable insights into the mechanisms that regulate lactation and support the development of precision nutrition and management strategies to improve productivity and metabolic health in dairy goats [9].

We hypothesized that differences in milk production between high-production (HP) and low-production (LP) Sapera goats would be reflected by distinct serum metabolic signatures, particularly in pathways related to energy metabolism, lipid utilization, amino acid metabolism, and lactation-associated metabolic adaptation. Therefore, the present study aimed to investigate the serum metabolomic profiles of HP and LP Sapera goats using liquid chromatography-high resolution mass spectrometry (LC-HRMS). By integrating multivariate statistical analyses, biomarker identification, metabolite enrichment analysis, and Kyoto Encyclopedia of Genes and Genomes (KEGG) pathway topology analysis, this study sought to identify key metabolites and metabolic pathways associated with differences in milk production. The findings of this study are expected to provide new insights into the metabolic regulation of lactation and contribute to the development of metabolite-based biomarkers and nutritional strategies to enhance productivity in dairy goat production systems.

## MATERIALS AND METHODS

### Ethical approval

All procedures involving animals and biological sample collection were reviewed and approved by the Ethics Committee of the Faculty of Animal Science, Universitas Hasanuddin, Makassar, Indonesia (Approval No. 015/UN4.12/EC/IV/2026). The study was conducted in accordance with institutional guidelines and national regulations governing the ethical use of animals in research. Blood samples were collected by trained personnel using aseptic techniques to minimize pain, stress, and discomfort to the animals. Throughout the experimental period, the goats were maintained under standard management and feeding conditions, and all efforts were made to ensure animal welfare and reduce unnecessary handling during sample collection and processing. No invasive procedures other than routine jugular venipuncture were performed, and no animals were euthanized for the purpose of this study.

### Study period and location

The study was conducted from January to April 2026 at the Laboratory of Animal Nutrition, Faculty of Animal Science, Universitas Hasanuddin, Makassar, Indonesia, and the Corpora Science Research Laboratory, Yogyakarta, Indonesia. All laboratory analyses and metabolomic profiling procedures were performed under standardized conditions to minimize environmental variations that could affect metabolic profiles.

### Study design

This study was designed to compare and characterize the metabolomic profiles of Sapera goats classified into two production groups. A total of 20 lactating Sapera goats were allocated into two balanced groups comprising 10 HP goats and 10 LP goats. The classification of HP and LP animals, together with animal metadata including parity, days in milk, age, body weight, body condition score, and general health status, followed the records established in our previous study on milk yield efficiency in Sapera dairy goats under tropical conditions [10]. In the present study, emphasis was placed on serum LC-HRMS-based metabolomic profiling and pathway analysis to identify discriminant metabolites and metabolic pathways associated with the HP and LP phenotypes. The classification of animals was based on their milk production performance during lactation. Goats with higher milk yield were categorized into the HP group, whereas those with lower milk yield were assigned to the LP group. All animals were maintained under similar feeding and management conditions to minimize environmental factors that could influence metabolic profiles.

### Milk production recording

Milk production was recorded to classify Sapera goats into different production groups. Daily milk yield was measured using a standardized milking procedure [11]. Milk produced by each goat was collected and measured during each milking session using a graduated measuring container, and the total daily milk yield was calculated by summing the milk obtained during morning and afternoon milking.

To obtain representative production values, milk production records were evaluated over three lactation periods. The average milk yield of each animal was calculated across these lactation periods. Animals with consistently higher average milk yield were categorized into the HP group, whereas animals with lower milk yield were classified into the LP group.

### Blood sampling and serum preparation

Blood samples were collected in the morning, within the same sampling window and under similar management conditions, before the main feeding period to minimize variation associated with diurnal rhythms and recent feed intake. Samples were collected individually from each goat and were not pooled. Following centrifugation, serum samples were visually inspected, and samples exhibiting obvious hemolysis were excluded from further metabolomic analysis.

Blood samples were collected aseptically from the jugular vein using sterile disposable 21G needles (Terumo®, Tokyo, Japan) and vacuum blood collection tubes without anticoagulant (Vacutainer®, BD, Franklin Lakes, NJ, USA). Blood samples were allowed to clot at 5°C for approximately 30 min, then centrifuged at 3,000 rpm (1,500–2,000 × *g*) for 10-15 min in a refrigerated centrifuge (Eppendorf® 5810R, Eppendorf, Hamburg, Germany) to separate serum from the clot. The resulting serum was carefully transferred into sterile 1.5-mL microcentrifuge tubes (Eppendorf) using sterile pipette tips (Axygen®, Corning Inc., Corning, NY, USA) [11].

### Metabolomic analysis

Serum metabolomic profiling was performed using LC-HRMS following established metabolomics workflows [11]. Metabolites were extracted using a methanol-based protein precipitation method. Briefly, 100 μL of each serum sample was transferred into 1.5-mL microcentrifuge tubes (Eppendorf) and mixed with prechilled 80% methanol (LC-MS grade; Thermo Fisher Scientific™, Waltham, MA, USA) by vigorous vortexing using a vortex mixer (VWR®, Radnor, PA, USA). Samples were incubated on ice for 5 min and centrifuged at 15,000 × *g* for 20 min at 4°C using a refrigerated microcentrifuge (Eppendorf® 5427R, Eppendorf, Hamburg, Germany). The resulting supernatant was collected and diluted with LC-MS-grade water (Thermo Fisher Scientific) to obtain a final methanol concentration of 53%. The solution was transferred into a fresh tube and centrifuged again at 15,000 × *g *for 20 min at 4°C to remove residual particulates. The final supernatant was transferred into LC-MS vials (Thermo Scientific™, Thermo Fisher Scientific, Waltham, MA, USA) and subjected to LC-MS/MS analysis.

Chromatographic separation was performed using a Thermo Scientific™ Vanquish™ Horizon ultra-high-performance liquid chromatography system (Thermo Fisher Scientific) equipped with a binary pump and an Accucore™ C18 column (100 mm × 2.1 mm internal diameter, 2.6 μm particle size; Thermo Fisher Scientific) maintained at 40°C. The mobile phases consisted of LC-MS-grade water containing 0.1% formic acid and LC-MS-grade acetonitrile containing 0.1% formic acid (Thermo Fisher Scientific). The mobile phase was delivered at a flow rate of 0.3 mL/min over a total run time of 25 min. The gradient elution program started at 5% organic phase, increased linearly to 90% over 16 min, was maintained at 90% for 4 min, and returned to 5% until 25 min for column re-equilibration. The injection volume was 5 μL.

Mass spectrometric detection was carried out using a Thermo Scientific™ Orbitrap™ Exploris 240 high resolution mass spectrometer (Thermo Fisher Scientific) operating in full MS/data-dependent MS2 acquisition mode with polarity switching in both positive and negative electrospray ionization modes. Data acquired in both ionization modes were processed using the same Compound Discoverer workflow. Features from both modes were annotated based on accurate mass, retention time alignment, MS/MS spectral matching, and database-supported identification. When the same metabolite was detected in both ionization modes, annotations with higher confidence, superior MS/MS spectral matching, and stronger database support were prioritized to avoid duplicate metabolite reporting. Full MS scans were acquired at a resolution of 60,000 full width at half maximum across an m/z range of 67-1000 with a mass tolerance of 5 ppm, whereas MS2 spectra were acquired at a resolution of 30,000 full width at half maximum using stepped normalized collision energies of 30, 50, and 70.

Raw LC-MS data were processed using Thermo Scientific™ Compound Discoverer™ software version 3.5 (Thermo Fisher Scientific). Peak detection, retention time alignment, and compound annotation were performed in accordance with standard metabolomics workflows. Raw data and processed features were inspected for peak quality, retention time consistency, mass accuracy, and MS/MS spectral quality. Metabolite identification was carried out by matching MS/MS spectra against the mzCloud spectral library (LC/MS Autoprocessed; LC/MS Reference). Structural prediction was further supported by using the SIRIUS software integrated with the KEGG and PubChem databases. Additional compound annotation was performed using ChemSpider-integrated resources, including the Human Metabolome Database, KEGG, PubChem, Chemical Entities of Biological Interest, ChEMBL, and FooDB. Mass list searches were also conducted using Human Metabolome Database version 5 mass lists and the Natural Products Atlas (release 2024_09) to improve metabolite annotation confidence. Features were retained for further analysis when they exhibited acceptable peak quality, mass accuracy within 5 ppm, and reliable database-supported annotation.

Pooled quality control samples and technical injection replicates were not included in the present LC-HRMS workflow; therefore, quality control-based coefficient of variation filtering and batch-effect correction were not applied.

### Bioinformatic analysis

Bioinformatic analysis followed the general workflow described by Yusuf* et al.* [12]. The processed feature table generated from Thermo Scientific™ Compound Discoverer™ software version 3.5 was used for downstream statistical and bioinformatic analyses. Before statistical interpretation, metabolite features were filtered based on annotation confidence, peak quality, MS/MS matching scores, mass accuracy (≤5 ppm), and database verification against mzCloud, KEGG, PubChem, and the Human Metabolome Database. Features with poor annotation confidence, inconsistent peak detection, or unreliable spectral matching were excluded from further analysis. Identified compounds were annotated using their corresponding KEGG identifiers to ensure standardized metabolite reporting and compatibility with KEGG-based pathway analysis.

The curated metabolite intensity table was imported into MetaboAnalyst 6.0 for statistical analysis. Data were normalized to reduce technical variation among samples, then log-transformed and scaled before multivariate analysis. Missing values, when present, were handled using the missing-value processing function in MetaboAnalyst, in which low-abundance values were replaced with a small-value imputation approach. The normalized dataset was subsequently used for multivariate and univariate statistical analyses.

Comparative metabolomic analyses were performed between HP and LP goats. Principal component analysis (PCA) was applied to evaluate clustering patterns, intrinsic variability, and potential outliers among samples. Subsequently, partial least squares-discriminant analysis (PLS-DA) was performed to identify metabolites contributing to discrimination between HP and LP groups, and model performance was evaluated using coefficients of determination and predictive capability values.

Differential metabolites between groups were identified using a volcano plot analysis that integrated fold change and statistical significance thresholds. Statistical significance was evaluated using Student's t-test or the Mann-Whitney U test when the assumption of normality was not satisfied. Metabolites with p < 0.05 and variable importance in projection scores >1.0 were considered significant contributors to group separation.

Metabolite annotation confidence was reported according to the Metabolomics Standards Initiative framework. Because authentic chemical standards were not used for confirmation, none of the discriminant metabolites were assigned to Level 1. The 15 key discriminant metabolites were classified as putatively annotated metabolites corresponding to Level 2 based on accurate mass, MS/MS spectral matching, and database-supported annotation using mzCloud, KEGG, PubChem, the Human Metabolome Database, ChemSpider-integrated resources, and SIRIUS-assisted structural prediction.

Hierarchical clustering analysis was conducted and visualized using heatmaps to illustrate relative metabolite abundance patterns across samples and groups. Receiver operating characteristic analysis was performed on selected discriminant metabolites to assess their ability to distinguish HP from LP goats. The results were used to support the biomarker potential of the selected metabolites while recognizing that further validation in larger independent populations is required before diagnostic application.

Finally, functional interpretation of differential metabolites was performed using KEGG pathway enrichment and pathway topology analyses in MetaboAnalyst 6.0 to identify metabolic pathways significantly associated with differences in milk production.

## RESULTS

### Multivariate analysis of metabolomic profiles

**Figure 1:**
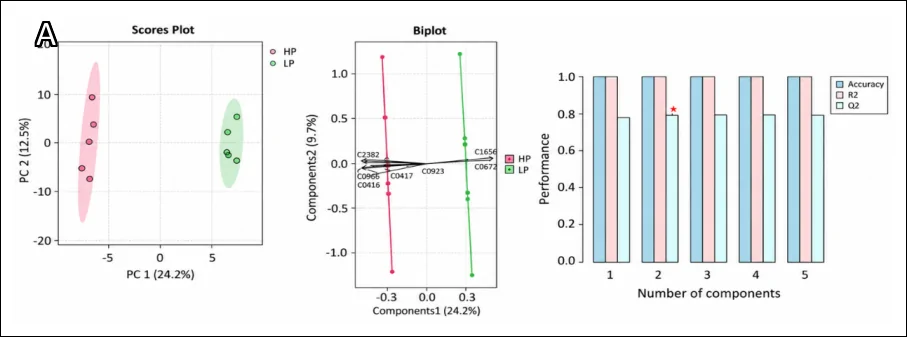
Multivariate analysis of metabolomic profiles in HP and LP Sapera goats. (A) PCA score plot showing sample clustering. (B) PLS-DA biplot illustrating group separation and contributing metabolites. (C) Cross-validation performance of the PLS-DA model across components.

Multivariate statistical analyses were performed to evaluate metabolic differences between HP and LP Sapera goats. PCA was first conducted as an unsupervised approach to visualize the overall variation in serum metabolomic profiles. As shown in Figure 1A, the PCA score plot demonstrated clear separation between the HP and LP groups along the first principal component. The first principal component explained 24.2% of the total variance, whereas the second explained 12.5%. This clustering pattern indicated that the metabolic profiles of the two production groups were distinct.

To further investigate group discrimination, PLS-DA was performed. The PLS-DA score plot (Figure 1B) showed clear separation between HP and LP samples along the first component, indicating distinct metabolic signatures associated with milk production levels. The loading vectors in the biplot indicated that several metabolites contributed strongly to the separation between the two groups.

The predictive performance of the PLS-DA model was evaluated using cross-validation. As illustrated in Figure 1C, the model achieved a high classification accuracy of nearly 1.0. The model fitness reached 1.0, indicating excellent explanation of data variance, whereas the predictive capability reached 0.79, indicating good predictive reliability. 

### Differential metabolites between HP and LP goats

**Figure 2:**
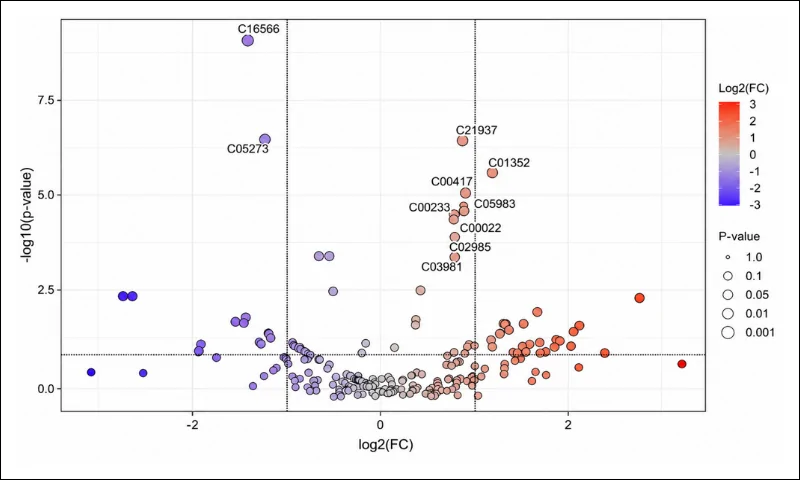
Volcano plot of differential metabolites between high-production and low-production Sapera goats.

To identify metabolites associated with differences in milk production, a volcano plot analysis was performed using fold change and statistical significance thresholds. The volcano plot (Figure 2) illustrates the distribution of metabolite features based on log2 fold change and −log10(p-value). Metabolites located on the right side of the plot represent compounds upregulated in the HP group, whereas metabolites on the left side represent compounds enriched in the LP group. Differential metabolites were initially screened for statistical significance and fold change patterns, and the final candidate discriminant metabolites were selected from those consistently contributing to group separation in the multivariate analyses.

Several metabolites showed significant differences between the two production groups (Table 1). Among the candidate discriminant metabolites, N-acetylneuraminic acid (C01352), 2-oxoadipic acid (C04438), fumarate (C00417), pyruvate (C00022), β-hydroxybutyric acid (C05983), glycerol 3-phosphate (C03981), 3-hydroxy-3-methylglutaric acid (C02985), N-carbamoyl-L-aspartate (C00233), and L-aminoadipic acid (C05267) showed higher abundance in the HP group. In contrast, sphinganine (C16566), cystathionine (C05273), phytosphingosine (C15767), sphingosine (C21963), cystathionine-related metabolites (C05269), and ceramide-related intermediates (C21937) were relatively enriched in the LP group.

Notably, sphinganine (C16566) showed the strongest statistical significance (−log10[p] = 9.06), followed by cystathionine (C05273) and N-acetylneuraminic acid (C01352), indicating that these metabolites contributed substantially to the metabolic differences observed between HP and LP Sapera goats. The identified differential metabolites were subsequently used to evaluate biomarkers and metabolic pathways.

### Key metabolites associated with milk production differences

**Figure 3:**
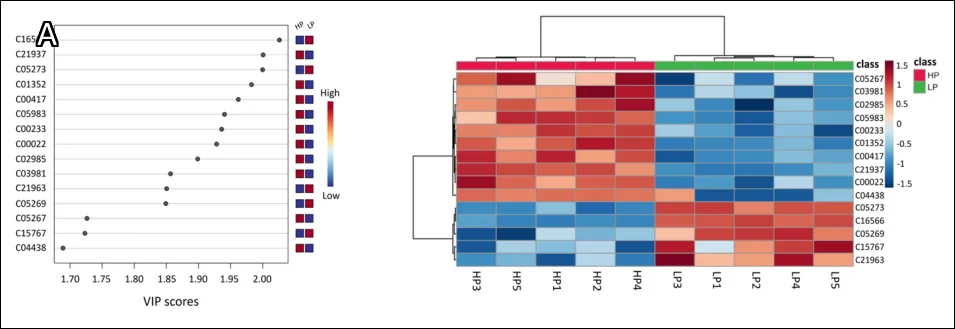
Key metabolites associated with milk production differences. (A) Variable importance in projection scores derived from the PLS-DA model showing the top metabolites contributing to discrimination between HP and LP Sapera goats. (B) Hierarchical clustering heatmap showing the relative abundance patterns of the top metabolites across samples. Red indicates higher relative abundance, whereas blue indicates lower abundance.

To identify metabolites that contributed most strongly to discrimination between HP and LP goats, variable importance in projection (VIP) analysis was performed on the PLS-DA model. Metabolites with VIP scores >1.0 were considered influential variables in group separation. The final panel of 15 candidate discriminant metabolites was selected based on their contribution to group separation, statistical significance, and biological relevance to lactation-related metabolic pathways. The top metabolites, ranked by VIP scores, are shown in Figure 3A.

Among the identified metabolites, sphinganine, ceramide-related intermediates, cystathionine, N-acetylneuraminic acid, and fumarate showed the highest VIP scores, indicating that these compounds contributed substantially to the metabolic differences between HP and LP groups. The distribution pattern indicated that several metabolites exhibited contrasting abundance profiles between the two production groups.

To further visualize the relative abundance patterns of these key metabolites across samples, hierarchical clustering analysis was performed and presented as a heatmap (Figure 3B). The heatmap revealed a clear clustering pattern separating HP and LP samples into distinct groups. Metabolites such as N-acetylneuraminic acid, fumarate, β-hydroxybutyric acid, N-carbamoyl-L-aspartate, and 3-hydroxy-3-methylglutaric acid showed higher abundance in the HP group, whereas cystathionine, sphinganine, cystathionine-related metabolites, phytosphingosine, and sphingosine were relatively enriched in the LP group. This clustering pattern confirms the metabolic distinction between HP and LP goats and highlights metabolites that may be associated with differences in milk production performance.

**Table 1:** Differential metabolites between high-production and low-production Sapera goats.

**KEGG ID**	**FC**	**log2(FC)**	**raw.pval**	**−log10(p)**
C16566	0.3759	−1.4116	8.77E−10	9.0569
C05273	0.42701	−1.2276	3.26E−07	6.4866
C01352	2.2732	1.1847	1.68E−06	5.7745
C04438	6.7157	2.7475	0.002927	2.5336
C01159	0.16053	−2.6391	0.0030748	2.5122
C00497	0.15012	−2.7358	0.0030942	2.5095
C00402	3.1749	1.6667	0.006687	2.1748
C05444	0.37024	−1.4335	0.009914	2.0038
C02220	0.34301	−1.5437	0.01294	1.8881
C00074	0.36457	−1.4557	0.013639	1.8652
C05840	2.4737	1.3067	0.013974	1.8547
C00033	2.5162	1.3312	0.014119	1.8502
C06427	2.8646	1.5183	0.01414	1.8496
C01384	2.4989	1.3213	0.014833	1.8288
C01056	4.3253	2.1128	0.015631	1.8060
C01879	2.5763	1.3653	0.019991	1.6992
C00712	4.1441	2.0510	0.022034	1.6569
C00134	2.4129	1.2707	0.024447	1.6118
C17646	2.4057	1.2664	0.025488	1.5937
C01157	0.4375	−1.1927	0.02582	1.5880
C04487	0.4389	−1.1880	0.026551	1.5759
C04442	0.44449	−1.1698	0.034058	1.4678
C12455	3.6514	1.8685	0.035024	1.4556
C00089	2.2600	1.1763	0.036372	1.4392
C00222	3.7416	1.9037	0.037734	1.4233
C01832	3.2305	1.6918	0.042048	1.3763
C00455	0.40952	−1.2880	0.042816	1.3684
C00064	3.0001	1.5850	0.046549	1.3321
C00636	0.41452	−1.2705	0.047242	1.3257
C16533	0.26639	−1.9084	0.049568	1.3048
C00037	4.0666	2.0238	0.050649	1.2954
C00412	2.8520	1.5120	0.053273	1.2735
C01013	3.6050	1.8500	0.053288	1.2734
C03046	2.2774	1.1874	0.053533	1.2714
C05266	3.3799	1.7570	0.069573	1.1576
C05161	0.26173	−1.9339	0.070993	1.1488
C00249	2.9188	1.5454	0.072359	1.1405
C16513	2.6638	1.4135	0.074276	1.1292
C08323	5.2095	2.3812	0.076216	1.1180
C02262	3.2420	1.6969	0.076661	1.1154
C05265	2.7452	1.4569	0.078212	1.1067

Positive log2(FC) values indicate higher abundance in HP goats, whereas negative log2(FC) values indicate higher abundance in LP goats. KEGG = Kyoto Encyclopedia of Genes and Genomes, FC = Fold change.

### Biomarker candidate performance

**Figure 4:**
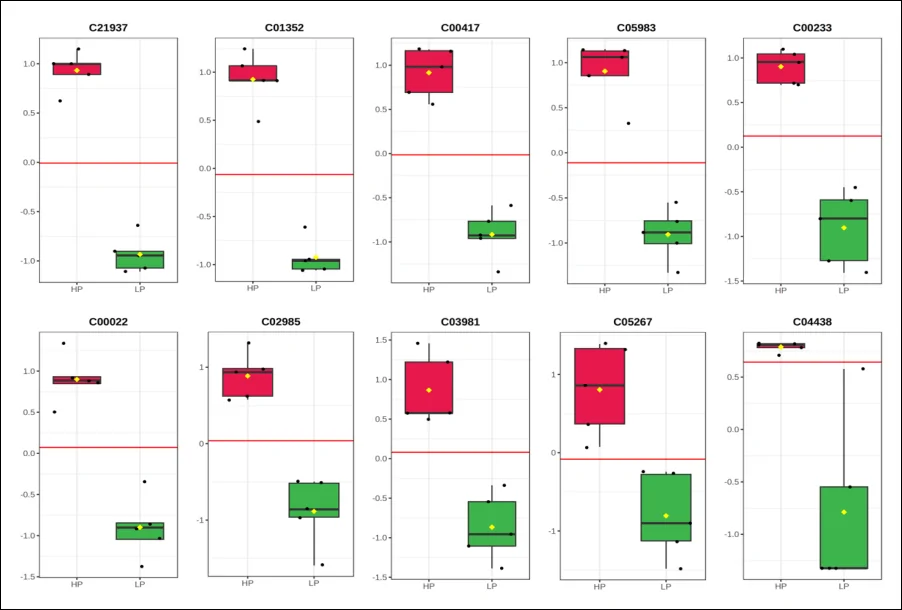
Boxplots of differential metabolites with higher abundance in high-production Sapera goats than in low-production Sapera goats.

**Figure 5:**
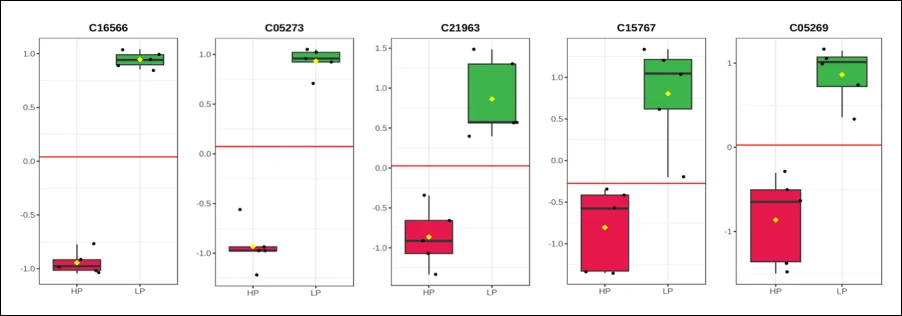
Boxplots of differential metabolites with higher relative abundance in low-production Sapera goats than in high-production Sapera goats.

The relative abundance patterns of selected differential metabolites were further examined to characterize metabolic differences between HP and LP Sapera goats (Figures 4 and 5). Several metabolites, including ceramide-related intermediates, N-acetylneuraminic acid, fumarate, β-hydroxybutyric acid, N-carbamoyl-L-aspartate, pyruvate, 3-hydroxy-3-methylglutaric acid, glycerol 3-phosphate, L-aminoadipic acid, and 2-oxoadipic acid, exhibited higher abundance in the HP group. These metabolites consistently showed elevated normalized intensities in HP samples, indicating their association with metabolic processes related to increased milk production.

In contrast, sphinganine, cystathionine, sphingosine, phytosphingosine, and cystathionine-related metabolites displayed higher abundance in the LP group. The abundance distributions of these metabolites clearly separated LP from HP animals, indicating metabolic alterations associated with lower milk production performance. These differences confirm the presence of distinct metabolic signatures between HP and LP Sapera goats.

### Metabolite set enrichment analysis

**Figure 6:**
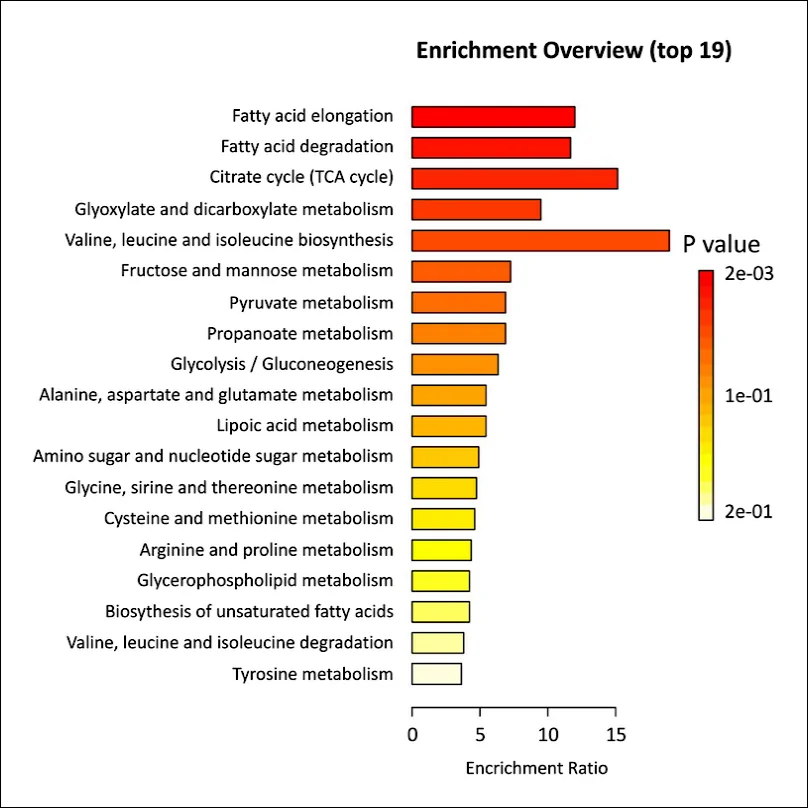
Metabolite set enrichment analysis of differential metabolites between high-production and low-production Sapera goats

Metabolite set enrichment analysis was conducted to identify metabolic functions associated with the differential metabolites detected between HP and LP Sapera goats (Figure 6; Table 2). The enrichment results revealed several metabolite sets associated with lipid, energy, and amino acid metabolism.

**Table 2:** Metabolite set enrichment analysis of differential metabolites between high-production and low-production Sapera goats.

**Metabolite set**	**Total**	**Expected**	**Hits**	**Raw p**	**Holm p**	**False discovery rate**
Fatty acid elongation	39	0.250	3	0.00154	0.124	0.0672
Fatty acid degradation	39	0.257	3	0.00166	0.133	0.0672
Citrate cycle (TCA cycle)	20	0.132	2	0.00696	0.550	0.188
Glyoxylate and dicarboxylate metabolism	32	0.211	2	0.0174	1.000	0.353
Valine, leucine and isoleucine biosynthesis	8	0.0527	1	0.0516	1.000	0.836
Fructose and mannose metabolism	21	0.138	1	0.130	1.000	1.000
Pyruvate metabolism	22	0.145	1	0.136	1.000	1.000
Propanoate metabolism	22	0.145	1	0.136	1.000	1.000
Glycolysis / Gluconeogenesis	24	0.158	1	0.148	1.000	1.000
Alanine, aspartate and glutamate metabolism	28	0.184	1	0.170	1.000	1.000
Lipoic acid metabolism	28	0.184	1	0.170	1.000	1.000
Amino sugar and nucleotide sugar metabolism	31	0.204	1	0.187	1.000	1.000
Glycine, serine and threonine metabolism	32	0.211	1	0.192	1.000	1.000
Cysteine and methionine metabolism	33	0.217	1	0.198	1.000	1.000
Arginine and proline metabolism	35	0.230	1	0.208	1.000	1.000
Glycerophospholipid metabolism	36	0.237	1	0.214	1.000	1.000
Biosynthesis of unsaturated fatty acids	36	0.237	1	0.214	1.000	1.000
Valine, leucine and isoleucine degradation	40	0.263	1	0.235	1.000	1.000
Tyrosine metabolism	42	0.276	1	0.245	1.000	1.000

Among the enriched metabolite sets, fatty acid elongation and fatty acid degradation showed the strongest enrichment signals, each containing three matched metabolites with the lowest raw p-values (p = 0.00154 and p = 0.00166, respectively). These metabolite sets exhibited the highest enrichment ratios, indicating a prominent role of fatty acid metabolism in the metabolic differences between HP and LP animals.

Other enriched metabolite sets included the TCA cycle and glyoxylate and dicarboxylate metabolism, suggesting alterations in central carbon metabolism. Several amino acid-related metabolite sets were also detected, including valine, leucine, and isoleucine biosynthesis; glycine, serine, and threonine metabolism; cysteine and methionine metabolism; and arginine and proline metabolism. Overall, the enrichment analysis indicates that lipid, central energy, and amino acid metabolism contribute to the metabolic differences observed between HP and LP Sapera goats.

### KEGG pathway analysis

**Figure 7:**
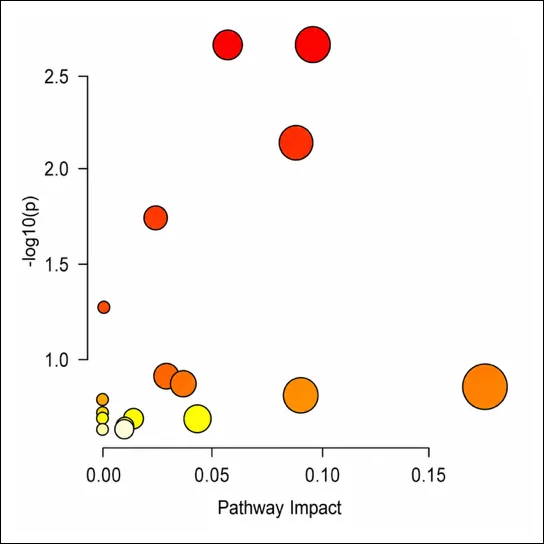
Kyoto Encyclopedia of Genes and Genomes pathway analysis of differential metabolites associated with milk production differences between high-production and low-production Sapera goats.

KEGG pathway analysis integrated pathway enrichment results with pathway topology information, allowing identification of pathways with both statistical significance and biological impact (Figure 7; Table 3). The analysis identified several pathways associated with metabolic differences between HP and LP Sapera goats.

**Table 3:** Kyoto Encyclopedia of Genes and Genomes pathway analysis of differential metabolites between high-production and low-production Sapera goats.

**Pathway name**	**Match status**	**p** **-value**	**−log(p)**	**Holm p**	**False discovery rate**	**Impact**
Fatty acid degradation	3/39	0.0015454	2.8110	0.12363	0.061814	0.06358
Fatty acid elongation	3/39	0.0015454	2.8110	0.12363	0.061814	0.10791
Citrate cycle (TCA cycle)	2/20	0.0066348	2.1782	0.51752	0.17693	0.09637
Glyoxylate and dicarboxylate metabolism	2/32	0.01662	1.7794	1.00000	0.33240	0.03000
Valine, leucine and isoleucine biosynthesis	1/8	0.050351	1.2980	1.00000	0.80562	0.00000
Fructose and mannose metabolism	1/20	0.12161	0.91504	1.00000	1.00000	0.03313
Propanoate metabolism	1/22	0.13300	0.87613	1.00000	1.00000	0.04103
Pyruvate metabolism	1/23	0.13865	0.85807	1.00000	1.00000	0.19137
Glycolysis or Gluconeogenesis	1/26	0.15540	0.80855	1.00000	1.00000	0.09785
Alanine, aspartate and glutamate metabolism	1/28	0.16640	0.77885	1.00000	1.00000	0.00000
Lipoic acid metabolism	1/28	0.16640	0.77885	1.00000	1.00000	0.00000
Cysteine and methionine metabolism	1/33	0.19334	0.71368	1.00000	1.00000	0.00000
Glycine, serine and threonine metabolism	1/34	0.19863	0.70195	1.00000	1.00000	0.00000
Arginine and proline metabolism	1/36	0.20913	0.67959	1.00000	1.00000	0.00000
Glycerophospholipid metabolism	1/36	0.20913	0.67959	1.00000	1.00000	0.01497
Biosynthesis of unsaturated fatty acids	1/36	0.20913	0.67959	1.00000	1.00000	0.04545
Valine, leucine and isoleucine degradation	1/40	0.22974	0.63876	1.00000	1.00000	0.01084
Tyrosine metabolism	1/42	0.23986	0.62003	1.00000	1.00000	0.00000
Amino sugar and nucleotide sugar metabolism	1/42	0.23986	0.62003	1.00000	1.00000	0.01063

Among these pathways, fatty acid degradation and fatty acid elongation exhibited the strongest enrichment signals, with the lowest p-values (p = 0.0015) and relatively high pathway impact values of 0.0636 and 0.1079, respectively. These results highlight the important role of lipid metabolism in the metabolic differentiation between HP and LP animals. The matched metabolites contributing to the enriched pathways were interpreted based on KEGG pathway mapping of the selected differential metabolites. Lipid-related pathways, including fatty acid elongation and degradation, were primarily supported by lipid-associated discriminant metabolites, whereas the TCA cycle and carbohydrate metabolism pathways were supported by central carbon metabolism intermediates such as fumarate and pyruvate.

In addition, pathways related to central energy metabolism, including the TCA cycle and glyoxylate and dicarboxylate metabolism, were identified with notable pathway impact values. Other pathways associated with carbohydrate and amino acid metabolism were also detected, including glycolysis or gluconeogenesis, pyruvate metabolism, propionate metabolism, and several amino acid metabolic pathways.

Overall, KEGG pathway topology analysis indicates that lipid metabolism, central carbon metabolism, and amino acid metabolism represent key metabolic processes underlying differences in milk production between HP and LP Sapera goats.

## DISCUSSION

### Metabolic signatures associated with milk production performance

This study identified a panel of 15 discriminant metabolites that clearly differentiated HP and LP Sapera goats, suggesting their potential as candidate metabolic biomarkers associated with milk production performance. These metabolites included pyruvate (C00022), N-carbamoyl-L-aspartate (C00233), fumarate (C00417), N-acetylneuraminic acid (C01352), 3-hydroxy-3-methylglutaric acid (C02985), glycerol 3-phosphate (C03981), 2-oxoadipic acid (C04438), L-aminoadipic acid (C05267), β-hydroxybutyric acid (C05983), cystathionine (C05273), sphinganine (C16566), phytosphingosine (C15767), sphingosine (C21963), ceramide-related intermediates (C21937), and cystathionine-related metabolites (C05269). These metabolites displayed clear differences in abundance between HP and LP animals, indicating that milk production performance is associated with systematic metabolic remodeling rather than random biochemical variability. In metabolomics studies, metabolites that consistently discriminate phenotypic groups often represent key metabolic nodes integrating multiple physiological processes, making them strong candidates for biomarker discovery [13, 14].

### Central carbon metabolism and energy production

Several metabolites enriched in HP goats were closely associated with central carbon metabolism and energy production, particularly pyruvate and fumarate. Pyruvate is a central intermediate linking glycolysis, gluconeogenesis, and mitochondrial respiration and therefore serves as a key metabolic junction regulating cellular energy metabolism [15]. Higher pyruvate abundance in HP goats may reflect differences in glycolytic activity and carbon substrate availability associated with lactation performance. Similarly, fumarate is an intermediate of the TCA cycle and reflects mitochondrial metabolic activity. Increased fumarate abundance may indicate altered central carbon metabolism between HP and LP goats and is consistent with the high energetic demand of lactation [16, 17]. However, serum metabolite abundance alone cannot confirm mitochondrial flux or determine whether these metabolic differences are a cause or consequence of higher milk production. Because lactation is one of the most energy-demanding physiological states in mammals, efficient mitochondrial metabolism is essential for maintaining sustained milk synthesis [18].

### Nucleotide metabolism and glycoprotein biosynthesis

Metabolites such as N-carbamoyl-L-aspartate and N-acetylneuraminic acid highlight the involvement of nucleotide metabolism and glycoprotein biosynthesis in lactation physiology. N-carbamoyl-L-aspartate participates in the de novo pyrimidine biosynthesis pathway and plays an important role in cellular proliferation and biosynthetic activity [19]. Increased levels of this metabolite may reflect enhanced cellular metabolic activity in the mammary gland during high milk production. N-acetylneuraminic acid, commonly referred to as sialic acid, is a key component of glycoproteins and glycolipids involved in cell signaling, immune regulation, and glycosylation processes [20]. Glycosylated molecules are important components of milk proteins and cellular membranes in mammary tissue, suggesting that altered sialic acid metabolism may reflect physiological adaptations that support milk synthesis [21].

### Lipid metabolism and membrane remodeling

Several metabolites identified in this study were associated with lipid metabolism and membrane remodeling, including 3-hydroxy-3-methylglutaric acid, glycerol 3-phosphate, 2-oxoadipic acid, L-aminoadipic acid, and β-hydroxybutyric acid. Lipid metabolism is a fundamental component of lactation biology because fatty acids and lipid-derived molecules provide both energy substrates and structural components required for milk fat synthesis [22]. β-hydroxybutyric acid, in particular, is a ketone body produced during fatty acid oxidation and reflects hepatic lipid metabolism and energy balance [23]. Increased levels of lipid-related metabolites may reflect greater lipid mobilization and utilization associated with the energetic requirements of lactation in HP goats [24]. Alternatively, these changes may represent systemic metabolic responses to higher milk output rather than direct mechanisms that drive production.

### Amino acid metabolism

Additional metabolites, including cystathionine, cystathionine-related metabolites, and amino acid-derived intermediates such as L-aminoadipic acid, highlight the involvement of amino acid metabolism in the metabolic differences between HP and LP goats. Amino acids serve as essential substrates for milk protein synthesis and participate in metabolic signaling pathways regulating nutrient utilization and metabolic homeostasis [25]. Alterations in amino acid metabolism may therefore reflect metabolic adjustments that support protein synthesis and nitrogen utilization during lactation [26].

### Sphingolipid-related metabolic changes

The identification of sphingolipid-related metabolites, including phytosphingosine, sphinganine, sphingosine, and ceramide-related intermediates, further suggests the involvement of lipid signaling pathways in metabolic adaptation to lactation. Sphingolipids play important roles in membrane structure, cell signaling, and metabolic regulation [27]. In lactating animals, membrane lipid turnover is particularly relevant because mammary epithelial cells undergo active secretory activity and continuous remodeling of cellular and milk fat globule membranes. Sphingolipid intermediates may also be linked to stress- and inflammation-related signaling, which can influence mammary gland function and metabolic adaptation during lactation. These molecules participate in lipid signaling pathways that regulate cellular stress responses, energy metabolism, and membrane remodeling [28]. Thus, the altered abundance of sphingolipid intermediates in this study may reflect differences in membrane lipid turnover, cellular signaling, or inflammation-related metabolic responses associated with lactation performance [29].

### Enriched metabolic pathways

The biological significance of these metabolites becomes clearer when examined through metabolite set enrichment analysis. The enrichment analysis revealed that the discriminatory metabolites converged into several interconnected metabolic functions, particularly fatty acid elongation, fatty acid degradation, and central carbon metabolism pathways. Fatty acid metabolism plays a central role in lactation physiology because fatty acids provide both energy substrates and structural components for milk fat synthesis [30]. Efficient lipid mobilization and oxidation enable lactating animals to meet the high energetic demands of continuous milk production [31]. The enrichment of fatty acid metabolic pathways suggests that lipid metabolism was an important systemic feature distinguishing HP and LP goats. This may reflect differences in lipid mobilization, oxidation, or nutrient partitioning associated with lactation performance.

Central carbon metabolism pathways, particularly the TCA cycle, were also identified as important metabolic processes distinguishing HP and LP goats. The TCA cycle integrates carbohydrate, lipid, and amino acid metabolism while generating adenosine triphosphate and biosynthetic precursors required for cellular function [32]. The involvement of the TCA cycle may reflect differences in mitochondrial energy metabolism and substrate oxidation between HP and LP goats [33].

Carbohydrate metabolism pathways, including glycolysis/gluconeogenesis, pyruvate metabolism, and propanoate metabolism, were also detected in the pathway analysis. In ruminants, propanoate produced during ruminal fermentation serves as the primary precursor for hepatic gluconeogenesis, ultimately providing glucose required for lactose synthesis in the mammary gland [34]. Because lactose synthesis largely determines milk volume through osmotic regulation of milk secretion, efficient glucose metabolism is critical for sustaining high milk yield [35].

### KEGG pathway topology interpretation

KEGG pathway topology analysis further reinforced the importance of lipid metabolism and central carbon metabolism in the metabolic differentiation between HP and LP goats. Pathways such as fatty acid degradation, fatty acid elongation, and the TCA cycle exhibited both strong statistical significance and notable pathway impact values. Pathway topology analysis evaluates not only the presence of metabolites within pathways but also their connectivity and influence within metabolic networks. Therefore, pathways with higher impact values may represent metabolically relevant hubs associated with differences between HP and LP goats [36].

### Comparison with previous studies

The metabolic patterns observed in this study are generally consistent with previous studies in dairy goats and cattle, which reported associations between lactation performance, metabolic efficiency, energy metabolism, lipid mobilization, amino acid metabolism, and ketone body-related pathways [16, 24]. In goats, previous metabolomic and physiological studies have shown that variation in milk yield or lactation status is associated with changes in mammary gland metabolism, mobilization of body energy reserves, and milk metabolite profiles [11, 24]. Similarly, studies in dairy cattle have identified metabolites in milk or blood as indicators of metabolic health and lactation performance, particularly during periods of high metabolic demand [5]. Compared with the previous integrative metabolomics and hormonal profiling study in Sapera goats [11], the present study focuses specifically on serum LC-HRMS-based discrimination between HP and LP groups and provides additional pathway-level interpretation through metabolite enrichment and KEGG pathway topology analyses. Therefore, the present findings extend current knowledge of dairy goat metabolomics by highlighting the combined involvement of fatty acid degradation, fatty acid elongation, central carbon metabolism, and sphingolipid-related metabolites in distinguishing HP and LP Sapera goats under tropical production conditions.

### Practical implications for precision nutrition

Beyond their biological significance, the metabolites identified in this study have important implications for precision nutrition strategies in dairy goat production systems. Precision nutrition aims to optimize nutrient supply to meet the metabolic requirements of individual animals, maximizing productivity while maintaining metabolic health [37]. The metabolic biomarkers identified in this study may therefore serve as valuable indicators of metabolic efficiency and nutrient utilization capacity in Sapera goats. Monitoring these metabolites could enable early identification of animals with superior metabolic adaptation to lactation, allowing targeted nutritional interventions to improve production efficiency [38]. Nutritional strategies targeting lipid metabolism, energy balance, and amino acid availability, such as improving dietary fatty acid balance, optimizing gluconeogenic precursor supply, and ensuring adequate amino acid availability, may be explored in future studies to determine whether they can improve lactation performance and metabolic stability in dairy goats [39].

### Study limitations and future perspectives

Despite these insights, several limitations should be considered when interpreting the results of this study. The relatively small sample size may limit statistical robustness and influence the predictive performance of the identified metabolites as candidate biomarkers [40]. In addition, the cross-sectional design and single sampling time point limit the ability to evaluate temporal metabolic changes across lactation. Environmental factors under tropical conditions, such as ambient temperature, humidity, and seasonal variation, may also influence feed intake, energy balance, and serum metabolite profiles. Another limitation is that milk composition traits, including milk fat, protein, and lactose contents, were not measured in the present study. Therefore, the metabolic differences observed between HP and LP goats should be interpreted as being associated with milk yield-based classification rather than milk composition or milk quality traits.

Another technical limitation is that pooled quality control samples and technical injection replicates were not included in the LC-HRMS workflow. Therefore, quality control-based coefficient of variation filtering and batch-effect correction could not be applied. Although data quality was assessed using peak quality, retention time alignment, mass accuracy, MS/MS spectral matching, and database-supported annotation, future metabolomics studies should include pooled quality control samples, technical replicates, and quality control-based filtering to improve analytical reproducibility and confidence. In addition, serum metabolomics reflects systemic metabolic responses and does not directly capture tissue-specific metabolic activity within organs directly involved in lactation, particularly the mammary gland and liver. Consequently, the observed metabolite changes represent systemic metabolic signatures rather than direct measurements of mammary gland or hepatic metabolic activity or metabolic flux within specific tissues [7].

Future studies should incorporate larger animal populations, longitudinal sampling across different lactation stages, milk composition analysis, pooled quality control samples, technical replicates, and integration with transcriptomic or proteomic analyses to provide deeper mechanistic insights into the metabolic regulation of milk production. Furthermore, targeted metabolite quantification and validation studies will be necessary to confirm the diagnostic and predictive utility of the candidate biomarkers identified in this study. Future studies integrating serum metabolomics with milk metabolomics, milk composition traits, hormonal profiling, enzyme activity, or metabolic flux analyses would provide stronger mechanistic insight into the relationship between metabolic pathways, milk yield, and milk quality in Sapera goats.

Overall, the findings of this study demonstrate that variation in milk production between HP and LP Sapera goats is associated with coordinated metabolic remodeling across lipid, central carbon, and amino acid metabolism. The 15 candidate metabolites identified in this study represent key intermediates in these metabolic networks and may serve as metabolic biomarkers that reflect lactation efficiency. These results provide new insights into the biochemical mechanisms underlying variation in milk production and highlight the potential of metabolomics for precision livestock nutrition and management strategies to improve productivity in dairy goat production systems.

## CONCLUSION

This study demonstrated that HP and LP Sapera goats exhibit distinct serum metabolomic signatures, indicating that variation in milk production is associated with coordinated metabolic remodeling rather than isolated biochemical changes. Multivariate analyses clearly discriminated between the two production groups and identified 15 candidate metabolites associated with differences in milk production performance. Metabolites related to central carbon metabolism, lipid metabolism, amino acid metabolism, and sphingolipid signaling, including pyruvate, fumarate, β-hydroxybutyric acid, glycerol 3-phosphate, N-acetylneuraminic acid, and sphinganine, contributed substantially to the metabolic differentiation between HP and LP animals. Enrichment and KEGG pathway analyses further revealed that fatty acid degradation, fatty acid elongation, the TCA cycle, carbohydrate metabolism, and amino acid metabolism are key metabolic pathways associated with differences in milk production.

These findings provide important practical implications for precision livestock management and precision nutrition in dairy goats. The identified metabolites may serve as candidate biomarkers for evaluating metabolic efficiency and lactation performance, and may facilitate the development of targeted nutritional strategies to optimize energy utilization, lipid metabolism, and amino acid availability, thereby improving milk production while maintaining metabolic health.

A major strength of this study lies in the integration of LC-HRMS-based metabolomic profiling with multivariate analysis, metabolite set enrichment analysis, and KEGG pathway topology analysis, providing a comprehensive systems-level understanding of metabolic adaptations associated with milk production in Sapera goats. In addition, this study provides one of the first detailed characterizations of serum metabolic signatures that distinguish HP and LP Sapera goats under tropical production conditions, thereby expanding current knowledge of dairy goat metabolomics.

Overall, the present findings demonstrate that lipid, central carbon, and amino acid metabolism are closely associated with lactation efficiency in Sapera goats. The identified metabolites represent promising candidate biomarkers of milk production performance and provide a foundation for future validation studies and the development of metabolomics-assisted nutritional and management strategies to enhance productivity and sustainability in dairy goat production systems.

## DATA AVAILABILITY

The datasets generated and analyzed during the current study are available from the corresponding author upon reasonable request.

## AUTHORS’ CONTRIBUTIONS

RI, AMD, and AN: Conceived, designed, and coordinated the study. RI, AMD, IUA and AN: Developed the study methodology, supervised the experimental procedures, and coordinated the research activities. FI, and RR: Designed data collection tools. RR, and AMA: Supervised field sampling, data collection, laboratory work, and data entry. AMD, AN, and FI: Statistical analysis and interpretation and drafted the manuscript. All authors have read and approved the final version of the manuscript.

## References

[1] Ibrahim NS, Jalil AR (2022). The effect of age on milk yield and milk composition in Saanen dairy goats. J Agric Sci Technol A.

[2] Gross JJ (2022). Limiting factors for milk production in dairy cows: perspectives from physiology and nutrition. J Anim Sci.

[3] Prates JA (2025). Heat stress effects on animal health and performance in monogastric livestock: physiological responses, molecular mechanisms, and management interventions. Vet Sci.

[4] Amara A, Frainay C, Jourdan F, Naake T, Neumann S, Novoa-Del-Toro EM (2022). Networks and graphs discovery in metabolomics data analysis and interpretation. Front Mol Biosci.

[5] Heirbaut S, Jing XP, Stefańska B, Pruszyńska-Oszmałek E, Buysse L, Lutakome P (2023). Diagnostic milk biomarkers for predicting the metabolic health status of dairy cattle during early lactation. J Dairy Sci.

[6] Guo Y, Wei Z, Zhang Y, Cao J (2024). Research progress on the mechanism of milk fat synthesis in cows and the effect of conjugated linoleic acid on milk fat metabolism and its underlying mechanism: a review. Animals.

[7] Hannan FM, Elajnaf T, Vandenberg LN, Kennedy SH, Thakker RV (2023). Hormonal regulation of mammary gland development and lactation. Nat Rev Endocrinol.

[8] Zhang J, Deng L, Zhang X, Cao Y, Li M, Yao J (2023). Multiple essential amino acids regulate mammary metabolism and milk protein synthesis in lactating dairy cows. Anim Feed Sci Technol.

[9] Zhao X, Tsermoula P, Khakimov B (2025). Applications of metabolomics in cow health assessment. Metabolomics.

[10] Islamiyati R, Azis IU, Amal I, Bahar MR, Sabil S, Santoso S (2025). Integrative metabolomics and hormonal profiling reveal biomarkers of milk yield efficiency in Sapera dairy goats under tropical conditions. Vet World.

[11] Boshoff M, Lopez-Villalobos N, Andrews C, Turner SA (2024). Modeling daily yields of milk, fat, protein, and lactose of New Zealand dairy goats undergoing standard and extended lactations. J Dairy Sci.

[12] Yusuf M, Diansyah AM, Sahiruddin S, Masturi M, Maulana T, Said S (2026). Integrated metabolomic and functional assessment of sexed frozen semen in Holstein Friesian bulls. Trop Anim Sci J.

[13] Brandl SJ, Lefcheck JS, Bates AE, Rasher DB, Norin T (2023). Can metabolic traits explain animal community assembly and functioning? Biol Rev.

[14] Bovo S, Ribani A, Fanelli F, Galimberti G, Martelli PL, Trevisi P (2025). Merging metabolomics and genomics provides a catalog of genetic factors that influence molecular phenotypes in pigs linking relevant metabolic pathways. Genet Sel Evol.

[15] Yiew NK, Finck BN (2022). The mitochondrial pyruvate carrier at the crossroads of intermediary metabolism. Am J Physiol Endocrinol Metab.

[16] Ribeiro DM, Palma M, Salvado J, Hernández-Castellano LE, Capote J, Castro N (2023). Goat mammary gland metabolism: an integrated omics analysis to unravel seasonal weight loss tolerance. J Proteomics.

[17] Álvarez-Delgado C (2022). The role of mitochondria and mitochondrial hormone receptors on the bioenergetic adaptations to lactation. Mol Cell Endocrinol.

[18] Meng M, Li X, Huo R, Ma N, Chang G, Shen X (2023). A high-concentrate diet induces mitochondrial dysfunction by activating the MAPK signaling pathway in the mammary gland of dairy cows. J Dairy Sci.

[19] Amos A, Amos A, Wu L, Xia H (2023). The Warburg effect modulates DHODH role in ferroptosis: a review. Cell Commun Signal.

[20] Zhang W, Zhou L, Huang X, Zhao X, Zheng H, Guo D (2025). Preventive and controlling effects of N-acetylneuraminic acid in regulating glucose and lipid metabolism disorders via the gut-liver axis in high-fat diet mice. Food Funct.

[21] Umar M, Ruktanonchai U, Makararpong D, Anal AK (2024). Enhancing immunity against pathogens through glycosylated bovine colostrum proteins. Food Rev Int.

[22] Tufarelli V, Colonna MA, Losacco C, Puvača N (2023). Biological health markers associated with oxidative stress in dairy cows during lactation period. Metabolites.

[23] Hu F, Liang C, Zhang X, Xu L, Wu C, Wan S (2025). From ketogenic metabolism to targeted therapeutics: current advances in β-hydroxybutyrylation. Front Immunol.

[24] de Oliveira TS, Rodrigues MT, Glória LS (2022). Mobilization of body energy reserves of alpine goats during early lactation in tropical conditions. Small Rumin Res.

[25] Batchu P, Naldurtiker A, Kouakou B, Terrill TH, McCommon GW, Kannan G (2022). Metabolomic exploration of the effects of habituation to livestock trailer and extended transportation in goats. Front Mol Biosci.

[26] Ye H, Langendijk P, Jaworski NW, Wu Y, Bai Y, Lu D (2022). Protein digestion kinetics influence maternal protein loss, litter growth, and nitrogen utilization in lactating sows. Front Nutr.

[27] Sasset L, Di Lorenzo A (2022). Sphingolipid metabolism and signaling in endothelial cell functions.

[28] Ji X, Chen Z, Wang Q, Li B, Wei Y, Li Y (2024). Sphingolipid metabolism controls mammalian heart regeneration. Cell Metab.

[29] He YR, Zhao HY, Li LL, Tan J, Wang Y, Zhao YC (2025). Metabolic lipid alterations in subclinical ketotic dairy cows: a multisample lipidomic approach.

[30] Veshkini A, Ceciliani F, Bonnet M, Hammon HM (2023). Effect of essential fatty acids and conjugated linoleic acid on the adaptive physiology of dairy cows during the transition period. Animal.

[31] Velasquez-Munoz A, Schuurmans EJ, Brester JL, Starken K, Abuelo A (2023). Association of maternal late-gestation lipid mobilization and their offspring's disease risk during the pre-weaned period and performance through first lactation: a cohort study in a dairy herd. Front Vet Sci.

[32] Arnold PK, Finley LW (2023). Regulation and function of the mammalian tricarboxylic acid cycle. J Biol Chem.

[33] Petrella L, Polito R, Catapano A, Santillo A, Ciliberti MG, Sevi A (2024). Goat milk supplementation modulates the mitochondrial metabolic flexibility and orexin-a levels influencing the inflammatory pattern in rats. Antioxidants.

[34] Wang G, Zhu Y, Feng D, Yao J, Cao Y, Deng L (2025). Hepatic gluconeogenesis and regulatory mechanisms in lactating ruminants: a literature review. Anim Res One Health.

[35] Hamon A, Guinard-Flament J, Costa A, Fischer A, Faverdin P, Gelé M (2025). Dynamics of the lactose content and other osmotic agents in milk throughout lactation according to the cow's parity. JDS Commun.

[36] Li B, Li Y, Tian W, Abebe BK, Raza SHA, Yu H (2025). Milk lipid regulation in dairy goats: a comprehensive review. Mol Biotechnol.

[37] Sonea C, Gheorghe-Irimia RA, Tapaloaga D, Gurau MR, Udrea L, Tapaloaga PR (2023). Optimizing animal nutrition and sustainability through precision feeding: a mini review of emerging strategies and technologies. Ann Valahia Univ Targoviste Agric.

[38] Abdelkrim AB, Ithurbide M, Larsen T, Schmidely P, Friggens NC (2023). Milk metabolites can characterise individual differences in animal resilience to a nutritional challenge in lactating dairy goats. Animal.

[39] Huang B, Khan MZ, Kou X, Chen Y, Liang H, Ullah Q (2023). Enhancing metabolism and milk production performance in periparturient dairy cattle through rumen-protected methionine and choline supplementation. Metabolites.

[40] Anwardeen NR, Diboun I, Mokrab Y, Althani AA, Elrayess MA (2023). Statistical methods and resources for biomarker discovery using metabolomics. BMC Bioinformatics.

